# Interaction between *Cucumber mosaic virus* 2b protein and plant catalase induces a specific necrosis in association with proteasome activity

**DOI:** 10.1007/s00299-016-2055-2

**Published:** 2016-09-22

**Authors:** Katsunori Murota, Hanako Shimura, Minoru Takeshita, Chikara Masuta

**Affiliations:** 1 Research Faculty of Agriculture, Hokkaido University, Kita-ku kita 9, Nishi 9, Sapporo, 060-8589 Japan; 2 Laboratory of Plant Pathology, Faculty of Agriculture, University of Miyazaki, Miyazaki, 889-2192 Japan

**Keywords:** *Cucumber mosaic virus*, Catalase, Ubiquitin–proteasome, 2b Protein *Arabidopsis thaliana*, Necrosis

## Abstract

**Key message:**

***Cucumber mosaic virus*** **(CMV) can induce a specific necrosis on** ***Arabidopsis*** **through the interaction between the CMV 2b protein and host catalase, in which the ubiquitin–proteasome pathway may be involved.**

**Abstract:**

We previously reported that the CMV 2b protein, the viral RNA silencing suppressor, interacted with the H_2_O_2_ scavenger catalase (*CAT3*), leading to necrosis on CMV-inoculated *Arabidopsis* leaves. We here confirmed that CMV could more abundantly accumulate in the *CAT3*-knockout mutant (cat3), and that *CAT3* makes host plants a little more tolerant to CMV. We also found that the necrosis severity is not simply explained by a high level of H_2_O_2_ given by the lack of *CAT3*, because the recombinant CMV, CMV-N, induced much milder necrosis in cat3 than in the wild type, suggesting some specific mechanism for the necrosis induction. To further characterize the 2b-inducing necrosis in relation to its binding to *CAT3*, we conducted the agroinfiltration experiments to overexpress *CAT3* and 2b in *N. benthamiana* leaves. The accumulation levels of *CAT3* were higher when co-expressed with the CMV-N 2b (N2b) than with CMV-Y 2b (Y2b). We infer that N2b made a more stable complex with *CAT3* than Y2b did, and the longevity of the 2b–*CAT3* complex seemed to be important to induce necrosis. By immunoprecipitation (IP) with an anti-ubiquitin antibody followed by the detection with anti-*CAT3* antibodies, we detected a higher molecular-weight smear and several breakdown products of *CAT3* among the IP-proteins. In addition, the proteasome inhibitor MG132 treatment could actually increase the accumulation levels of *CAT3*. This study suggests that the host proteasome pathway is, at least partially, responsible for the degradation of *CAT3*, which is manifested in CMV-infected tissues.

**Electronic supplementary material:**

The online version of this article (doi:10.1007/s00299-016-2055-2) contains supplementary material, which is available to authorized users.

## Introduction

Reactive oxygen species (ROS), such as hydrogen peroxide (H_2_O_2_) and O_2_^−^, are generated during numerous physiological processes, including photosynthesis, plant development, and resistance responses against pathogens. H_2_O_2_ serves as an important molecular messenger to induce a form of programmed cell death (PCD) and especially called as the hypersensitive reaction (HR) in plant–pathogen interactions. Catalase is one of the most important antioxidant enzymes that catalyze the decomposition of H_2_O_2_, thus playing a role in protecting cells from H_2_O_2_ toxicity. *Arabidopsis* has three catalase enzymes (Frugoli et al. 1996), and catalase 3 (*CAT3*) is the most abundantly expressed and controlled by a circadian rhythm. *CAT3* expression is enhanced with plant age and is accompanied by H_2_O_2_ accumulation in vascular bundles (Zimmermann et al. 2006; Hu et al. 2010). *CAT3* has been found to interact with several proteins, such as nucleoside diphosphate kinase, NDK1 (Fukamatsu et al. 2003), class 3 sucrose-nonfermenting 1-related kinase, SOS2 (Verslues et al. 2007), and LESION SIMULATING DISEASE1, LSD1 (Li et al. 2013), and even a viral protein, the 2b protein (2b) of *Cucumber mosaic virus* (CMV) (Inaba et al. 2011). The interactions between catalase and other proteins may cause the diverse effects on catalase’s function. For example, Zou et al. (2015) demonstrated that the interaction between *CAT3* and calcium-dependent protein kinase 8 (CPK8) enhanced *CAT3* activity to maintain H_2_O_2_ homeostasis in response to drought stress. On the other hand, some interactions cause functional disturbance of catalase resulting in the accumulation of H_2_O_2_ and subsequent cell death (i.e., necrosis); the interactions of *CAT3* with LSD1 and with 2b have been reported to be involved in necrosis on *Arabidopsis* (Inaba et al. 2011; Li et al. 2013).

CMV, the type member of the genus *Cucumovirus*, has a broad host range of more than 1000 plant species. It has a tripartite genome consisting of RNAs 1–3. RNAs 1 and 2 encode viral helicase and replicase, respectively, for viral replication, and RNA 3 encodes the viral movement protein 3a. RNA 4, a subgenomic RNA derived from the 3′ half of RNA 3, is the mRNA for the coat protein, while RNA 4A, a subgenomic RNA from RNA 2, encodes 2b (Ding et al. 1994). 2b is known as an RNA silencing suppressor (RSS) and also functions in viral cell-to-cell and long-distance movement (Ding et al. 1995; Ji and Ding 2001; Soards et al. 2002; Shi et al. 2003; Goto et al. 2007). In addition, 2b contains nuclear localization signals (NLSs) that are required for the manifestation of viral symptoms and for RSS activity (Lucy et al. 2000; Lewsey et al. 2009). We previously found a protein–protein interaction between 2b and *Arabidopsis* *CAT3*, which apparently causes H_2_O_2_ accumulation and subsequent necrosis in infected *Arabidopsis* leaves. The interaction between 2b and *CAT3* also dramatically changes the localization of *CAT3*, which is normally localized in the cytoplasm; *CAT3* was translocated to the nucleus in the presence of 2b (Inaba et al. 2011; Masuta et al. 2012).

Although catalase is well known to play an important role in regulating HR through the decomposition of H_2_O_2_ during plant–pathogen interactions, there are not many reports that describe the molecular details of the catalase-mediated pathways against viruses. For CMV in pepper plants, catalase activity was important for determining the degree of host susceptibility to CMV (Petrova et al. 2009). In addition, it was shown that CMV infection significantly induced catalase expression in squash plants (Havelda and Maule 2000). We also observed that 2b’s RSS activities were cancelled by a high level of *CAT3* expression in the protoplast experiment (Inaba et al. 2011), and that *CAT3*-overexpressing transgenic Col-0 lines showed the suppression of CMV multiplication until 7 dpi, although the levels of CMV reached those of the nontransgenic control plants at 14 dpi. Therefore, *CAT3* seems to play an antiviral role in CMV infection, but a role of *CAT3* in CMV tolerance of *Arabidopsis* still remains unknown. For the necrosis induction, we reasoned that *CAT3* and 2b were important, but we did not have any answer to explain the phenomenon that the necrosis severity greatly varied depending on the CMV strains and *Arabidopsis* ecotypes. Here, we further investigated the mechanism underlying the manifestation of necrosis symptoms observed in *Arabidopsis* infected with CMV. Our results of this study suggest that the stability of the *CAT3*-2b complex is important for the necrosis, and that the proteasome system is involved in degrading CAT and regulating the induction of necrosis in *Arabidopsis*.

## Materials and methods

### Plant materials and viruses

For *Arabidopsis thaliana*, ecotype Col-0 and the *CAT3*-knockout Col-0 mutant (cat3) were used in this study. *Arabidopsis* was grown in a growth chamber at 21 °C with 12 h photoperiod (150 μmol/m^2^/s). *Nicotiana benthamiana*, which were used for agroinfiltration, was grown at 23 °C with 16 h light/8 h dark. To create *CAT3*-complemented plants (*CAT3*/cat3), the homozygous cat3 plant (cat3/cat3, T-DNA insertion line) was transformed with the wild-type *CAT3* cDNA. We first PCR-amplified the cDNA covering the ORF using a primer pair (the forward primer, 5′-GGACTAGTATGGATCCTTACAAGTATCGTCC-3′ and the reverse primer, 5′-GCGGAGCTCCTAGATGCTTGGCCTGACGTTCAG-3′) based on the sequence in GenBank (accession no. NM_001035996). The cDNA was then inserted in the plant expression vector, pIG121-Hm, to create pIG21-*CAT3*. cat3 plants were transformed with pIG21-*CAT3* by the conventional floral dip method. T1 plants were selected for resistance to hygromycin and used for the subsequent inoculation experiments. CMV-Y infectious clones (pCY1, pCY2, and pCY3) (Suzuki et al. 1991) and the CMV-Y-based vectors of CMV-A1 (Otagaki et al. 2006) and CMV-H1 (Matsuo et al. 2007) were previously constructed. A1Ds and H1Ds were created by inserting the *Ds-Red2* gene into CMV-A1 or CMV-H1, respectively (Takeshita et al. 2012). CMV-N has the CMV-Y backbone, but it contains a different C-terminal of the 2b protein; CMV-N was coincidentally created by inserting a 100-bp DNA fragment into the CMV-A1 vector.

### Viral inoculation and fluorescence microscopy

The plasmids containing the full-length cDNAs of RNAs of CMV were transcribed in vitro. Leaves of 6-week-old plants of *N. benthamiana* were dusted with carborundum and rub-inoculated with the in vitro-transcribed RNAs. For *Arabidopsis*, 4-week-old plants were inoculated with the sap of infected tissues. Ds-Red2 fluorescence images were taken essentially according to Takeshita et al. (2012). In brief, red fluorescence of inoculated leaves of *Arabidopsis* Col-0 and cat3 was acquired using SMZ1500 (Nikon) with Ds-Red2 filter sets. For each inoculum, a set of four plants was used. Leaves of different plants were removed and used for imaging at 11 dpi.

### BiFC assay

The BiFC plasmid vectors for transient expression (Singh et al. 2009) were kindly supplied by Dr. S. Mano, National Institute for Basic Biology, Japan. The full-length cDNA of the *CAT3* gene of *Arabidopsis* was cloned in either pGWnG or pnGGW, while the 2b gene was cloned in either pcCGGW or pGWcCG. All constructs were created by the Gateway Technology (Invitrogen). To amplify the designed fusion genes from the constructs containing the inserts, PCR was conducted using the forward primer (T7 promoter sequence + the 5′ end sequence of the ORF for the N-terminal protein) and the reverse primer (oligo-dT of 66 T residues + the sequence of the 3′ nontranslated region just before the terminator). Capped RNAs were then in vitro-transcribed from the PCR products and subsequently co-transfected into *N. benthamiana* protoplasts as essentially described before (Shimura et al. 2008a, b).

### MG132 treatment

For the MG132 treatment, either healthy Col-0 leaves or CMV-Y-inoculated leaves at 2 dpi were detached from the basal part of the petiole, and the leaves were then transferred to glass tubes containing 50 μM MG132 (Sigma), which was originally dissolved in DMSO, and incubated at 21 °C with 12 h photoperiod for 3 days before protein extraction.

### Quantitative RT-PCR

Total RNA was isolated using the Trizol reagent (Invitrogen) essentially as described before (Kim et al. 2008). Total RNA (100 ng) was used for the first-strand cDNA synthesis by AMV reverse transcriptase (Nippon Gene). Quantitative PCR was performed using Universal SYBR Select Master Mix (Applied Biosystems) in a StepOne Real-Time PCR System (Applied Biosystems). The *Arabidopsis* tubulin gene (AtTub) was used as an internal control. Primer sets for each gene amplification were as follows: 5′-GAGGGAGCCATTGACAACATCTT-3′ and 5′-GCGAACAGTTCACAGCTATGTTCA-3′ (for AtTub), 5′-GCGCGTCGACGTTGACGTCGAGCACCAAC-3′ and 5′-CCATCGATTGGTCTCCTTTTGGAGGCC-3′ (for CMV).

### Agroinfiltration experiments

The plasmid construct of pBE2113:*CAT3*-FLAG with a FLAG tag sequence at the 3′ end has been already described (Inaba et al. 2011). In addition, the FLAG-*CAT3* with a FLAG tag at the 5′ end and the 2b gene of CMV-N (N2b) were inserted in the Ti plasmid vector pBE2113 in this study. The agroinfiltration was conducted according to Goto et al. (2007). *Agrobacterium* (KYRT1) culture containing each construct (FLAG-*CAT3*, *CAT3*-FLAG, Y2b, and N2b) was prepared to an optimal density (OD) at 600 of 1.0 and infiltrated in *N. benthamiana* leaves using a 1-ml syringe. Total proteins were extracted 3 days postinfiltration (dpi) and then subjected to Western blot analysis.

### Immunoprecipitation and Western blot analysis

Total protein was extracted from the inoculated leaves essentially as described before (Masuta et al. 1995). Immunoprecipitation was performed using Dynabeads protein G (Life Technologies) with anti-ubiquitin antibody (Abcam) according to the method essentially described by He and Kermode (2010). Western blots were probed using either anti-FLAG (Sigma) antibody or anti-*CAT3* antibodies (Inaba et al. 2011). The anti-*CAT3* antibodies can recognize the catalase(s) in *N. benthamiana* (at least NbCAT1) as well as *Arabidopsis* *CAT3*, because *CAT3* and NbCAT1 share 94 % amino-acid sequence similarity.

### Statistical analysis

Data were evaluated using Student’s *t* test. A *P* value of <0.05 was considered to be significant.

## Results

### CMV symptom, accumulation, and movement in *CAT3*-knockout mutant (cat3)

**Fig. 1:**
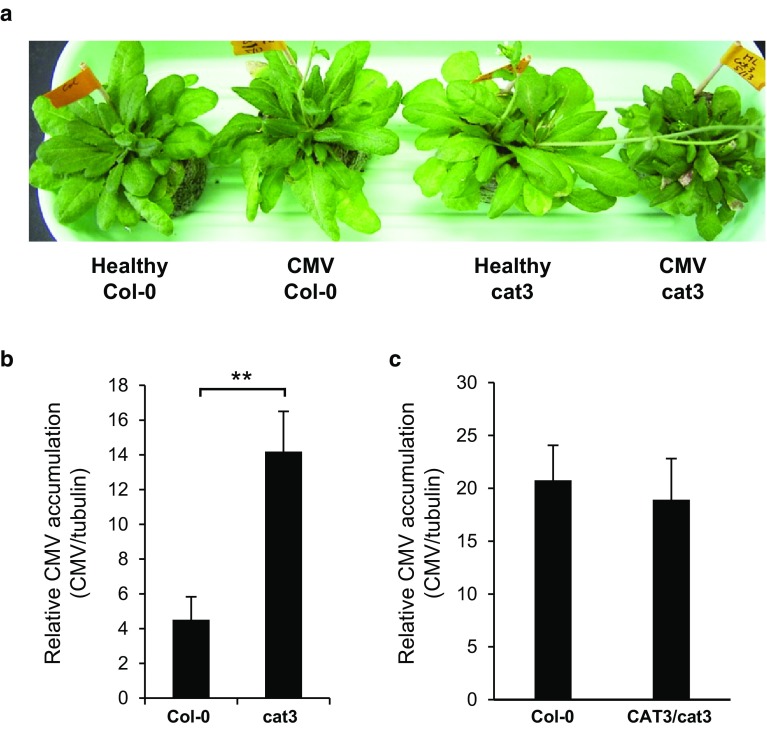
Viral symptom and accumulation in CMV-infected *Arabidopsis* cat3 (*CAT3*-knockout Col-0 mutant). **a** Stunting of *cat3* plant at 14 dpi with CMV. Healthy plants were mock-inoculated. **b** Levels of CMV accumulation in upper leaves of *cat3* plants at 14 dpi analyzed by quantitative RT-PCR. Values were normalized using the mRNA levels of the β-*tubulin* gene. The averages ±SD of three biological replicates are shown. Differences in means were evaluated by *t* test; *asterisks* indicate significance at the 0.01 level. **c** Levels of CMV accumulation in upper leaves of *CAT3*-complemented plants (*CAT3*/cat3). CMV accumulation was measured by quantitative RT-PCR at 10 dpi. The averages ±SD of three biological replicates are shown

We previously reported that the interaction between *CAT3* and CMV 2b causes necrosis accompanied with H_2_O_2_ accumulation, suggesting that the malfunction of *CAT3* induced H_2_O_2_-mediated cell death. We here examined the effects of depletion of *CAT3* from *Arabidopsis* on CMV symptom and accumulation using *CAT3*-knockout plants of Col-0 (cat3). The T-DNA insertion knockout mutant (SALK-092911) was obtained from the Arabidopsis Biological Resource Center (ABRC). We identified homozygous T-DNA insertion line from T4 plants using PCR. Compared with the wild-type control plants, the cat3 plants infected with CMV-Y at 14 dpi were somewhat more stunted (Fig. 1a) and showed weak necrosis; there was a little difference in visible necrotic appearance between wild type (Col-0) and cat3. We examined the catalase activities and found that the activities were one-third of the levels in the control even after CMV infection (Supplementary Fig. 1). These results suggest that the depletion of *CAT3* did not facilitate necrosis symptoms, and that 2b alone has an ability to essentially induce weak necrosis on *Arabidopsis* leaves.

**Fig. 2:**
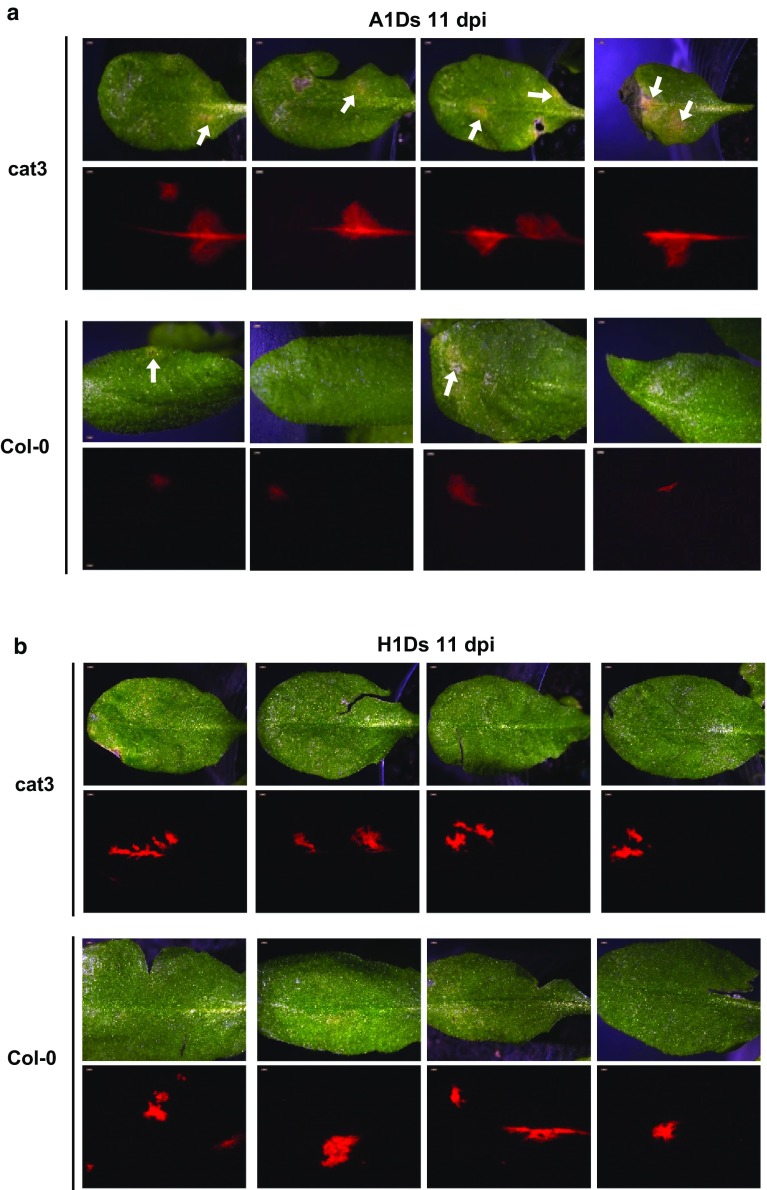
Localization of CMV in *Arabidopsis* Col-0 and cat3 plants. Spreads of A1Ds (**a**) or H1Ds (**b**) in the inoculated leaves of Col-0 and cat3 were observed under a fluorescence microscope at 11 dpi. Light micrographs (*upper*) and fluorescence micrographs (*lower*) are shown as a pair. *Red areas* indicate Ds-Red2 fluorescence and thus show the localization of CMV. A1Ds and H1Ds were created by inserting the *Ds-Red2* gene into CMV-Y-based vectors, CMV-A1 or CMV-H1, respectively. CMV-H1 does not have 2b. A1Ds induced necrotic spots (*arrows*), while no necrotic spots were detected on leaves infected with H1Ds (color figure online)

We previously reported that *CAT3* can weaken the RSS activity of 2b, and that CMV levels were reduced at least until 7 dpi in *CAT3*-overexpressing Col-0 (Inaba et al. 2011). In this study, we observed that CMV accumulation levels were significantly higher in cat3 plants at 14 dpi (Fig. 1b). To confirm that the susceptibility of cat3 to CMV was due to disruption of the *CAT3* gene, we complemented cat3/cat3 with the wild-type *CAT3* gene. We selected several hygromicin-resistant T1 plants, which produce certain levels of the *CAT3* gene transcript (Supplementary Fig. 2). When those T1 transformants were inoculated with CMV, there was no difference in CMV accumulation between WT and the *CAT3*-complemented cat3/cat3 (*CAT3*/cat3) (Fig. 1c). We also analyzed viral movement in cat3 plants by monitoring fluorescence in leaves inoculated with a recombinant CMV expressing the DS-Red2 protein (A1Ds) (Supplementary Fig. 3a; Takeshita et al. 2012). A1Ds induced weak necrotic spots on both Col-0 and cat3, and spread more rapidly in the inoculated leaves of cat3 than in the wild-type control (Fig. 2a). On the other hand, for the CMV-H1 vector (H1Ds) that does not have 2b, we did not find any difference in viral spread between WT and cat3, and any necrotic spots on the inoculated leaves (Fig. 2b). These results suggest that 2b is indispensable for necrosis induced by CMV, and that the presence of 2b can make CMV move more rapidly in cat3 than in wild-type. Taken together, to some extent, *CAT3* contributes to inhibiting CMV movement.

### Specific induction of necrosis through the interaction between 2b and *CAT3*

**Fig. 3:**
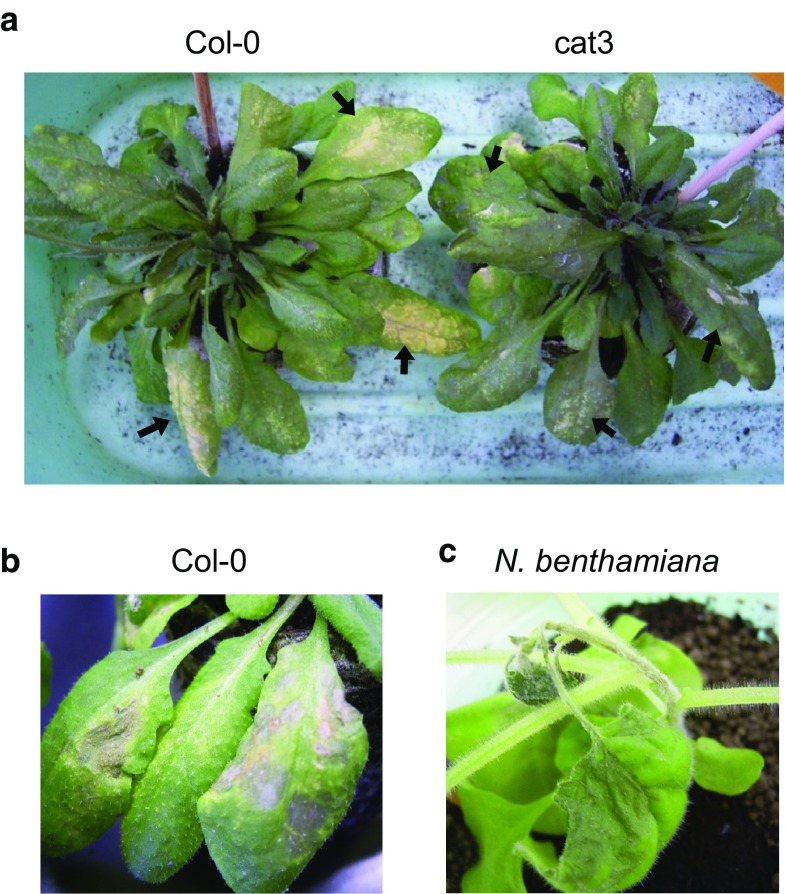
Necrotic symptoms on CMV-N-inoculated plants at 5 dpi. **a** CMV-N-inoculated *Arabidopsis* Col-0 and cat3. *Arrows*: inoculated leaves. **b** Close up of necrosis on Col-0 leaves inoculated with CMV-N. **c** Lethal systemic necrosis on *N. benthamiana* infected with CMV-N at 10 dpi

**Fig. 4:**
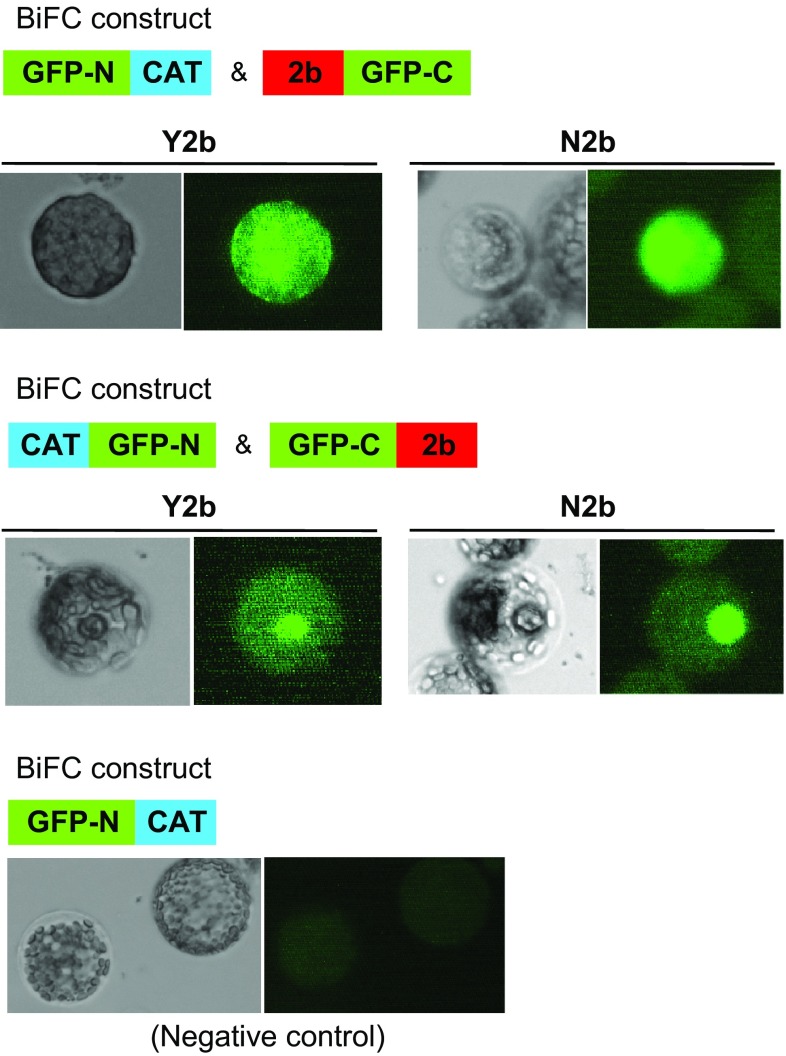
Interaction between 2b (either Y2b or N2b) and *CAT3* proteins detected by BiFC in protoplasts. *N. benthamiana* protoplasts were transfected with in vitro-transcribed RNA for BiFC, as schematically shown in the *upper panel*. GFP-C, the C-terminal half of GFP; GFP-N, the N-terminal half of GFP. 12 h after transfection, GFP fluorescence was observed by a fluorescence microscope and the *typical images* are shown; the *left side image* is for bright field and the right side is for GFP. Note that the GFP fluorescence was mainly localized possibly in the nucleus and also in the cytosol, and that GFP fluorescence was slightly stronger between N2b and *CAT3* than between Y2b and *CAT3*

In our previous experiments, we found that the recombinant CMV, Y1H2Y3, which has the CMV-Y backbone but contains HL2b derived from CMV-HL (Inaba et al. 2011), induced necrotic spots on infected *Arabidopsis* plants, which are more discrete than those induced by CMV-Y. In this study, we found that another recombinant CMV (CMV-N) could induce very severe necrosis that spread rapidly over the inoculated leaves. The 2b protein of CMV-N (N2b) has a nonviral C-terminal sequence (56 amino acids) (Supplementary Fig. 3b), which was coincidentally created by the insertion of a 100 bp-foreign fragment in the CMV-A1 vector. For *Arabidopsis*, CMV-N induced a very distinct, extensive necrosis on inoculated Col-0 leaves (Fig. 3a, b). However, when we inoculated CMV-N onto cat3 plants, we observed much milder necrosis (Fig. 3a), suggesting that the necrosis severity on CMV-inoculated *Arabidopsis* leaves is determined by the presence of *CAT3*; *CAT3* would be a modulator of the 2b-inducing necrosis. In addition to *Arabidopsis*, CMV-N induced not only severe necrosis on the inoculated leaves but also systemic lethal necrosis in *N. benthamiana* (Fig. 3c). To confirm whether N2b still has an ability to interact with *CAT3*, the BiFC assay was conducted by co-transfecting *N. benthamiana* protoplasts with two combinations of the N-terminal and C-terminal GFP constructs for BiFC. As shown in Fig. 4, we observed distinct GFP fluorescence derived from the interactions between N2b and *CAT3*, as is the case in the interaction between Y2b and *CAT3* (Inaba et al. 2011). In this assay, we noticed that GFP fluorescence might be localized possibly in the nucleus and to a lesser extent in the cytosol. These observations suggest that *CAT3* can interact with N2b like Y2b, and that 2b-inducing necrosis feature is determined by a specific combination of 2b and *CAT3*.

### CAT degradation through the ubiquitin–proteasome pathway depending on a different combination of 2b and *CAT3*

**Fig. 5:**
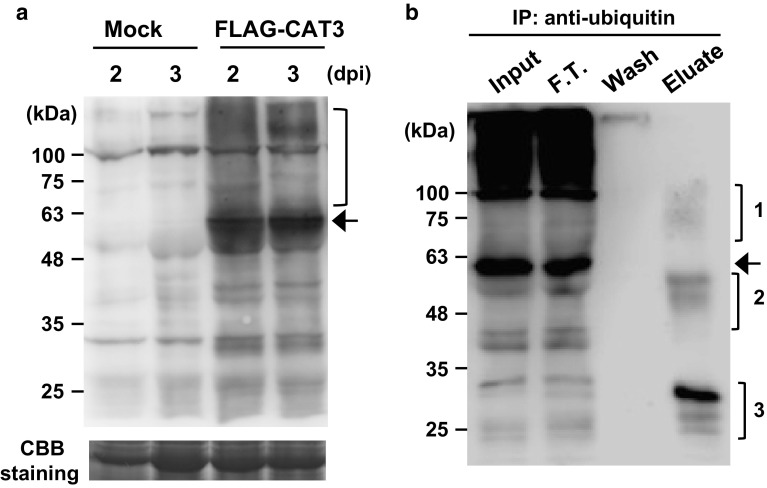
*CAT3* accumulation in leaves of *N. benthamiana* agroinfiltrated with the FLAG-*CAT3* construct. **a** Western blot analysis of protein extracts from agroinfiltrated leaves at 2 and 3 dpi using anti-FLAG antibody to detect *CAT3*. Coomassie brilliant blue (CBB) staining was used to monitor the equivalence of protein loading. *Bracket* shows FLAG-*CAT3* proteins with higher molecular weights. Mock, buffer-infiltrated. **b** Ubiquitination of the *CAT3* protein in leaves agroinfiltrated with the FLAG-*CAT3*. Protein extracts were prepared from the agroinfiltrated leaves expressing the FLAG-*CAT3* construct at 3 dpi. Immunoprecipitation was performed with anti-ubiquitin antibodies, and precipitated FLAG-*CAT3* proteins were detected with anti-FLAG antibody. Three size groups (*brackets 1–3*) of eluted proteins were detected; the strongest band is ~30 kDa. *Arrow* indicates FLAG-*CAT3* (~60 kDa)

To artificially reproduce such a symptom by co-expressing 2b and *CAT3*, we conducted overexpression experiments of the two proteins in *N. benthamiana* by the *Agrobacterium*-mediated transient expression assay. However, we eventually found that *CAT3* did not accumulate to an expected high level, but seemed to be degraded to some extent. We also detected a distinct higher molecular-weight smear above the 57-kDa band of *CAT3* using anti-FLAG antibody against FLAG-*CAT3* in the infiltrated tissues but not in the Mock (Fig. 5a). Because we suspected that the high molecular-weight smear might be due to ubiquitination of *CAT3*, we conducted immunoprecipitation (IP) with anti-ubiquitin antibodies and then detected *CAT3* with anti-*CAT3* antibodies. Our Western blot analysis revealed that the anti-ubiquitin IP fraction indeed contained *CAT3*, whose molecular weights are classified into three size groups (1–3) (Fig. 5b). Size 1 contained a smear, which should be the higher molecular weight ubiquitinated CAT proteins, and size 2 and 3 contained several bands for possible breakdown products of *CAT3*. In this Western blot, we were not able to detect any protein signals over 100 kDa in the IP fraction, while the input and flow-through lanes contained a strong smear >100 kDa. Heavily ubiquitinated, high molecular-weight CAT proteins may not have efficiently been precipitated with the anti-ubiquitin antibody under the nondenaturing conditions used.

**Fig. 6:**
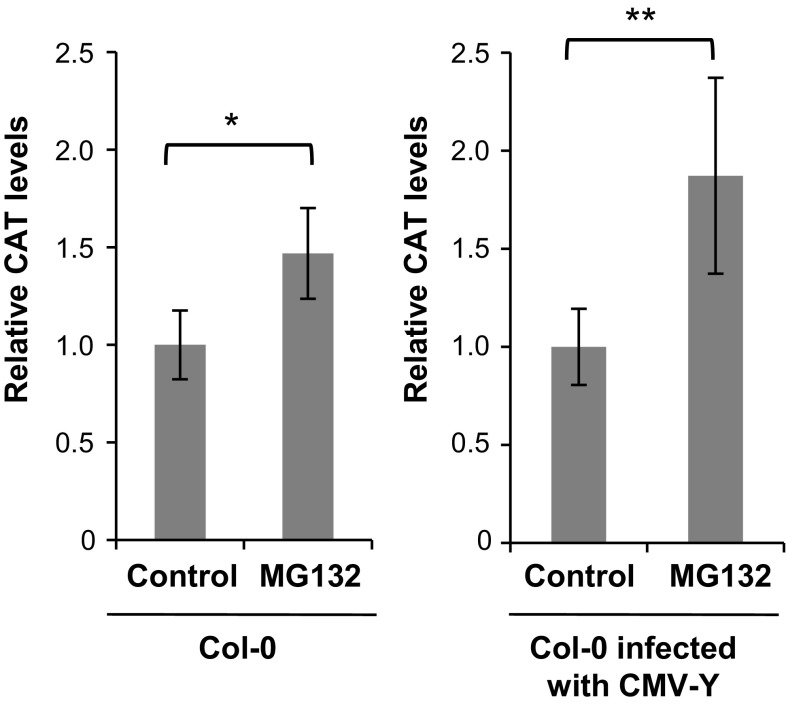
*CAT3* accumulation levels in Col-0 or CMV-Y-infected Col-0 after MG132 treatment. CMV-Y was inoculated 2 days before MG132 treatment. Leaves were treated for 3 days by MG132, and *CAT3* was detected by Western blots using anti-*CAT3* antibodies. The band intensity of *CAT3* was normalized by that of Rubisco large subunit visualized by Coomassie brilliant blue (CBB) staining. The *CAT3* level in nontreated control leaves (Control) was set to 1.0. Data are means of 3–4 independent replicates with SD. Differences in means were evaluated by *t* test; *one asterisk* and *two asterisk* indicate significance at the 0.05 and 0.01 levels, respectively. A representative image of a Western blot is shown in Supplementary Fig. 4

Because ubiquitination of protein is a signal for degradation through the proteasome, we inferred that *CAT3* might be degraded by the ubiquitin–proteasome pathway. To confirm the involvement of proteasome in the degradation of *CAT3*, we treated Col-0 leaves for 3 days with a proteasome inhibitor MG132 and then examined CAT levels by Western blot (Fig. 6). The *CAT3* band intensity was normalized by the Rubisco band intensity. When healthy leaves were treated with MG132, the CAT levels were approximately 1.3-fold increased (Fig. 6). When Col-0 leaves were inoculated with CMV-Y and those leaves were treated with MG132 at 2 dpi, a twofold increase in *CAT3* levels was detected compared with the untreated control (Fig. 6, Supplementary Fig. 4). These results suggest that proteasome activity is, at least partially, responsible for the *CAT3* degradation, which is promoted by CMV infection.

**Fig. 7:**
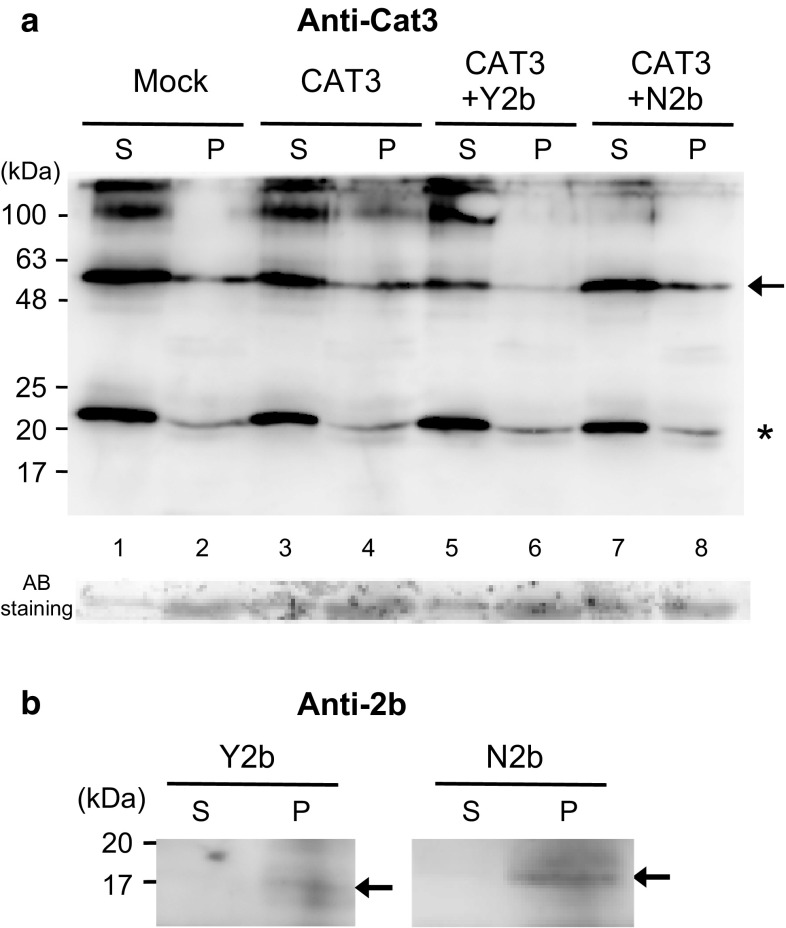
Degradation of *CAT3* protein in leaves agroinfiltrated with *CAT3*-FLAG and CMV 2b (Y2b or N2b). **a** Western blot analysis of the protein extracts from agroinfiltrated leaves at 3 dpi. *CAT3*-FLAG was detected using anti-*CAT3* antibodies. Protein samples were prepared as essentially described in Masuta et al. (1995). Briefly, leaf tissue was homogenized in 1 ml of PBS/Tween and centrifuged at 10000×*g* for 20 min to separate the supernatant (*S*) and pellet (*P*). After Laemmli’s sample buffer was added to each of *S* and P, the samples were subjected to SDS-PAGE. *Arrow* indicates both the endogenous tobacco catalases and *CAT3*-FLAG proteins, and asterisk shows a possible breakdown product derived from the catalases. The blot was stained with amido black (AB) to confirm the equivalence of protein loading. **b** Western blot with anti-2b antibodies. Y2b and N2b were detected on the same blot. *Arrows* indicate the 2b protein. The loading controls of* S* and* P lanes* of Y2b and N2b are equal to *lanes 5* and *6* (for Y2b) and *lanes 7* and *8* (for N2b) of AB staining in **a**, respectively

We next investigated whether 2b can affect *CAT3* degradation by the Western blot analysis following the transient overexpression of *CAT3* and 2b by agroinfiltration. The overexpression of *CAT3*-FLAG itself caused a slight decrease in the *CAT3* level compared with the Mock control (Fig. 7 lanes 1, 3). In contrast, the coexpression of *CAT3* and Y2b markedly reduced the *CAT3* levels (lanes 5, 6). On the other hand, the overexpression of N2b did not reduce the *CAT3* level as efficiently as Y2b did (lanes 7, 8). In this experiment, we also observed that Y2b was barely detected compared with the distinct band of N2b in the *Agrobacterium*-infiltrated tissues (Fig. 7), suggesting that Y2b was less stable than N2b. These results suggest that the degradation of Y2b and *CAT3* was simultaneously taken place when they were co-expressed but that in the combination of N2b and *CAT3*, they were more slowly degraded.

## Discussion

To analyze the interaction between 2b and *CAT3*, we previously produced transgenic Col-0 plants that overexpress the *CAT3* gene under the 35S promoter (Inaba et al. 2011). With difficulty, we finally obtained several *CAT3*-overexpressing transgenic lines, but they produced *CAT3* at most ~twofold more than in the wild type (Inaba et al. 2011). We assumed that when the accumulation levels of *CAT3* exceed a certain threshold, the *CAT3* protein levels must be lowered by a well-regulated mechanism, because the *CAT3* levels should be very critical to maintain the cellular redox balance. Based on the detection of the higher molecular weight, ubiquitinated smear band (above the 57 kDa) in the agroinfiltration experiment, we considered that the proteasome pathway regulated *CAT3* degradation. *CAT3* expression levels are drastically controlled by a circadian rhythm, in which the amplitude of the oscillations in *CAT3* mRNA accumulation is ~fivefold; the lowest peak is in the early morning (Zhong and McClung 1996). If *CAT3* functions according to the circadian rhythm and is regulated at the mRNA level, the synthesized protein should be quickly degraded. We thus consider that even in healthy Col-0 plants, *CAT3* levels must be reset by protein degradation along with the circadian rhythm. In fact, a proteasome inhibitor, MG132, treatment increased 1.3-fold *CAT3* accumulation compared to the untreated control, while the *CAT3* levels were increased ca. twofold by MG132 when plants were infected with CMV-Y (Fig. 6). These results provide the evidence that the accumulation levels of *CAT3* in the presence of CMV 2b are significantly affected by the ubiquitin–proteasome pathway.

As for the involvement of the proteasome in the regulation of cellular *CAT3* levels and association of programmed cell death with the generation of ROS, this study is not the first one. He and Kermode (2010) have already demonstrated that white spruce plants actually used the proteasome to control the levels of CAT (a homolog of *Arabidopsis* *CAT3*) during the seedling development. One important observation that we share with them is the finding of several proteins with lower molecular weights as breakdown products of CAT after MG132 treatment. As shown in Fig. 5, we also detected several lower molecular weights bands (20–30 kDa) in our Western blots, suggesting that those breakdown products of *CAT3* may have been generated through the same ubiquitin–proteasome pathway, as is the case for the white spruce CAT. When these results are considered together, it is likely that the proteasome-mediated *Arabidopsis* *CAT3* degradation plays an important role in viral symptom expression and host defense in CMV-infected plants.

Although we here described that interaction between 2b and *CAT3* promoted *CAT3* degradation probably by the ubiquitin–proteasome pathway, the phenomena that viral proteins can promote degradation of host factors through the proteasome system are not rare events (reviewed by Verchot 2014). As for the viral RSS-mediated degradation of a host factor, Chiu et al. (2010) demonstrated that P25, the RSS of PVX, could induce the degradation of host AGO1 via proteasomes by agroinfiltration in *N. benthamiana*. The P0 proteins of *Polerovirus* and *Enamovirus* have been also shown to target AGO1 for degradation (Baumberger et al. 2007; Fusaro et al. 2012). On the other hand, it is noteworthy that many viral RSSs are ubiquitinated to be targeted for proteasome degradation (reviewed by Alcaide-Loridan and Jupin 2012). Conversely, the HC-Pro protein, the RSS of *Papaya ringspot virus* (PRSV), can inhibit host proteasomes, enhancing viral infection (Sahana et al. 2012). Therefore, viral RSSs appear to be deeply involved in the ubiquitin–proteasome pathway during plant–virus interactions whether viruses use it to degrade cellular proteins for their own benefit or inhibit it to prevent them from being a target.

In this study, we demonstrated that *CAT3* would work as a modulator of the 2b-inducing necrosis, and that the catalase binding to 2b would be degraded through the proteasome pathway. Considering the catalase activity to erase H_2_O_2_ triggering HR, it is conceivable that the observed necrosis may be a simple result of catalase consumption by the interaction between 2b and *CAT3*. However, there are also some other explanations. For example, Li et al. (2013) previously demonstrated that the interaction between LSD (an important negative regulator of PCD) and *Arabidopsis* catalases (CATs) played an important role in pathogen-induced PCD, which requires the accumulation of salicylic acid. This LSD1-mediated PCD may be involved in the CMV-induced necrosis. Alternatively, based on the observation by He and Kermode (2010) that CAT was ubiquitinated and degraded by the proteasome, in white spruce just before extensive PCD occurred, we may raise another possibility that the 2b’s binding to *CAT3* can induce necrosis by a certain specific mechanism, which is not necessarily explained only by the catalase activity. This idea is consistent with our finding that the necrosis severity and development depend on the 2b’s sequences; we here assume the involvement of resistance gene (*R* gene)-mediated HR. Because the necrosis induced by CMV infection accompanied H_2_O_2_ generation and expression of the *PR* genes (Inaba et al. 2011), this necrosis could be regarded as an HR-like defense response. In addition, N2b could induce very severe necrosis (even lethal necrosis) not only in *Arabidopsis* but also in *N. benthamiana*, suggesting the involvement of systemic HR-like necrosis driven by an R gene, depending on the nature of the interaction between 2b and catalase. Several reports, indeed, suggest that the ubiquitin–proteasome pathway is deeply involved in HR-like cell death in host defense responses (Liu et al. 2002; Kim et al. 2003; Yang et al. 2006; Sadanandom et al. 2012). For example, gene silencing of the COP9 signalosome, a multiprotein complex involved in protein degradation via the ubiquitin–proteasome pathway compromised the *R* gene (*N* gene)-mediated HR on tobacco infected with *Tobacco mosaic virus* (TMV) (Liu et al. 2002). Similarly, silencing of an ACRE276, E3-ubiquitin ligase in the proteasome pathway resulted in loss of two HRs in tobacco: *N*-mediated HR to the Avr protein p50 of TMV and also *Cf9*-mediated HR to the Avr protein Avr 9(4) of *Cladosporium fulvum* (Yang et al. 2006). Although any *R* gene that can recognize CMV 2b has not been identified, we rather prefer the idea that a *CAT3*-2b complex may induce HR-like cell death in a putative *R* gene-mediated resistance, which is associated with the ubiquitin–proteasome pathway.

### **Author contribution statement**

KM, HS, and CM designed the experiments and wrote the paper. MT conducted the inoculation experiments using the CMV vector expressing Ds-Red.

## Electronic supplementary material

Below is the link to the electronic supplementary material.

Supplementary material 1 (PPTX 248 kb)

## Abbreviations

CAT: Catalase
CMV: Cucumber mosaic virus
H_2_O_2_: Hydrogen peroxide
HR: Hypersensitive reaction
PCD: Programmed cell death
ROS: Reactive oxygen species
