# Supplementary material for: Interaction between Cucumber mosaic virus 2b protein and plant catalase induces a specific necrosis in association with proteasome activity
Source: Plant Cell Rep. 2016 Sep 22;36(1):37–47. doi: 10.1007/s00299-016-2055-2 (PMC5206265; doi:10.1007/s00299-016-2055-2)
Supplement: Supplementary file 1 — Supplementary material 1 (PPTX 248 kb) [file 299_2016_2055_MOESM1_ESM.pptx]

## Slide 1
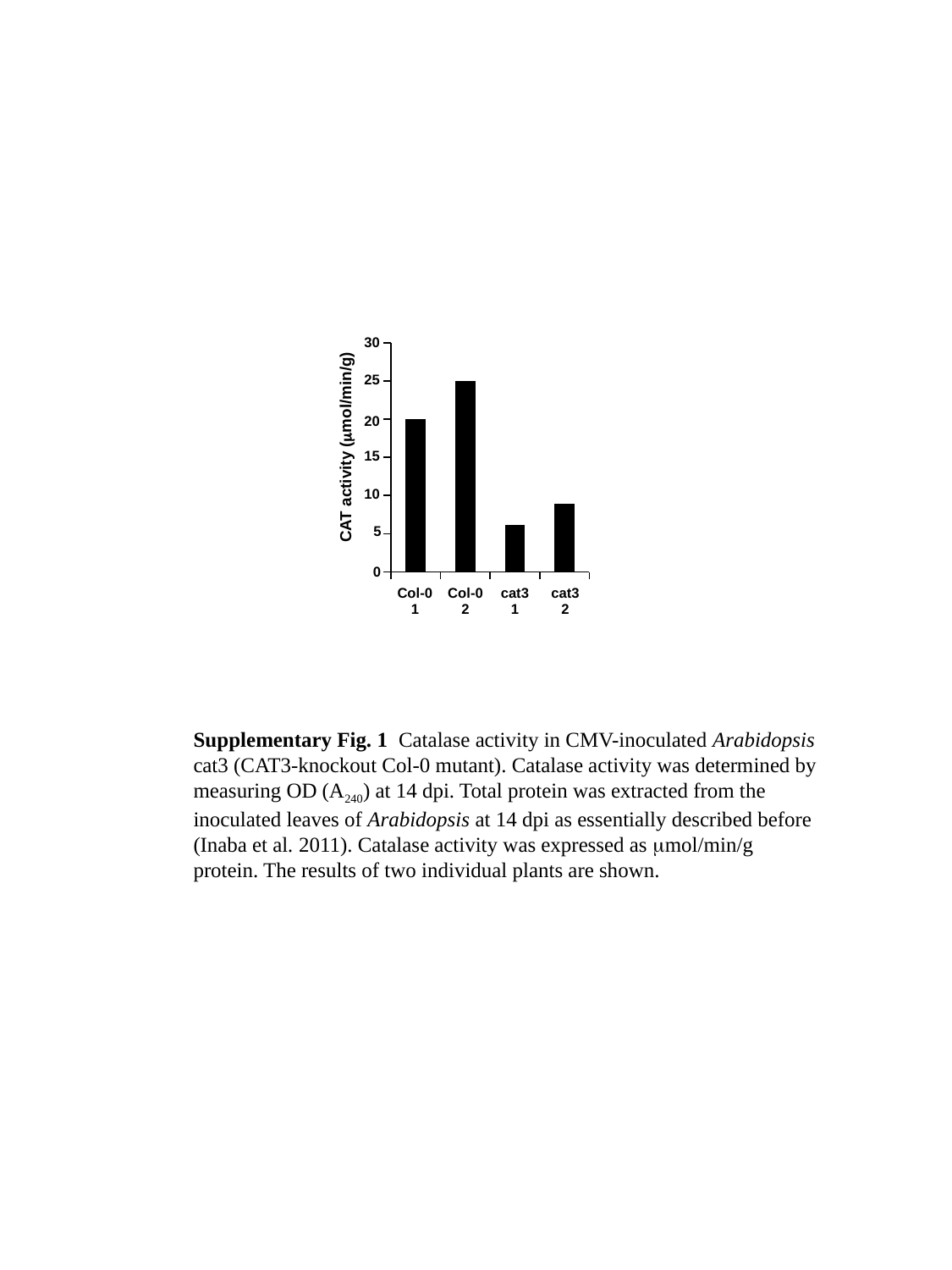

### Chart
| Category | |
|---|---|
| Col-1 | 20.0 |
| Col-2 | 25.0 |
| cat3-1 | 6.109999999999999 |
| cat3-2 | 8.89 |30
25
20
CAT activity (mmol/min/g)
15
10
5
0
Col-0
1
Col-0
2
cat3
1
cat3
2
Supplementary Fig. 1 Catalase activity in CMV-inoculated Arabidopsis cat3 (CAT3-knockout Col-0 mutant). Catalase activity was determined by measuring OD (A240) at 14 dpi. Total protein was extracted from the inoculated leaves of Arabidopsis at 14 dpi as essentially described before (Inaba et al. 2011). Catalase activity was expressed as mmol/min/g protein. The results of two individual plants are shown.

## Slide 2
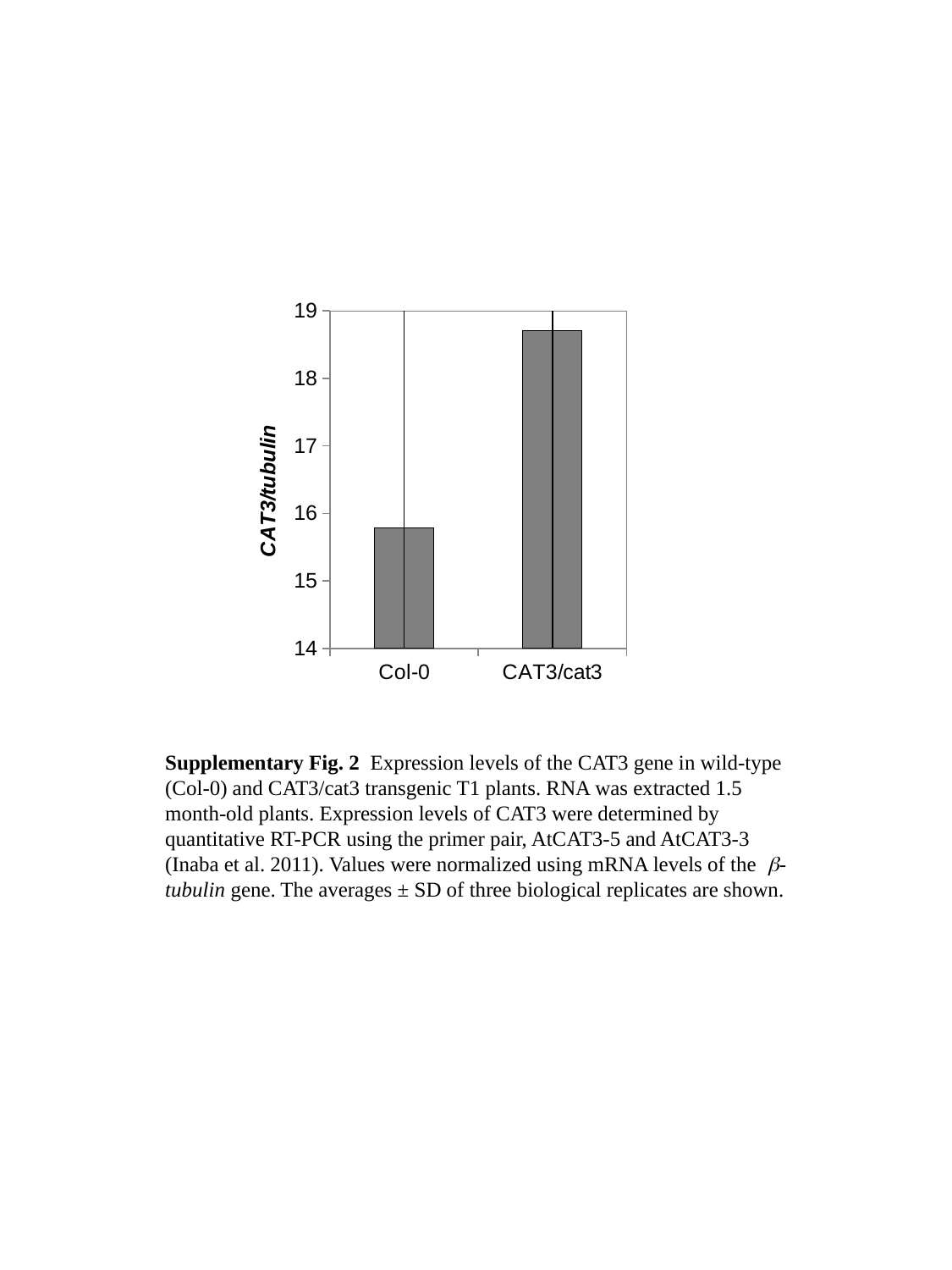

### Chart
| Category | |
|---|---|
| Col-0 | 15.78741868946025 |
| CAT3/cat3 | 18.70254032269718 |Supplementary Fig. 2 Expression levels of the CAT3 gene in wild-type (Col-0) and CAT3/cat3 transgenic T1 plants. RNA was extracted 1.5 month-old plants. Expression levels of CAT3 were determined by quantitative RT-PCR using the primer pair, AtCAT3-5 and AtCAT3-3 (Inaba et al. 2011). Values were normalized using mRNA levels of the b-tubulin gene. The averages ± SD of three biological replicates are shown.

## Slide 3
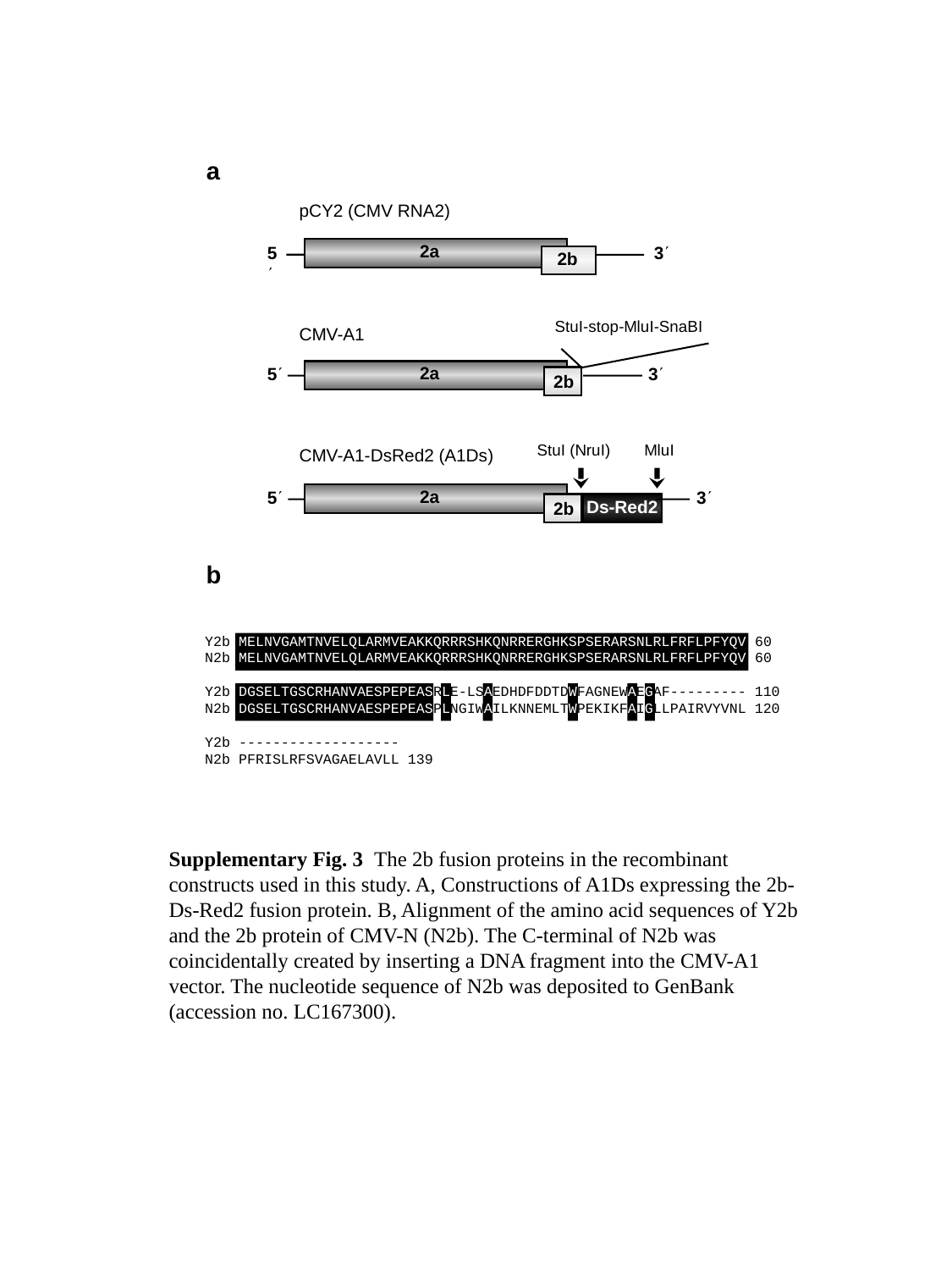

a
pCY2 (CMV RNA2)
2a
5′
3′
2b
StuI-stop-MluI-SnaBI
CMV-A1
2a
5′
3′
2b
StuI (NruI)
MluI
CMV-A1-DsRed2 (A1Ds)
2a
5′
3′
Ds-Red2
2b
b
Y2b MELNVGAMTNVELQLARMVEAKKQRRRSHKQNRRERGHKSPSERARSNLRLFRFLPFYQV 60
N2b MELNVGAMTNVELQLARMVEAKKQRRRSHKQNRRERGHKSPSERARSNLRLFRFLPFYQV 60
Y2b DGSELTGSCRHANVAESPEPEASRLE-LSAEDHDFDDTDWFAGNEWAEGAF--------- 110
N2b DGSELTGSCRHANVAESPEPEASPLNGIWAILKNNEMLTWPEKIKFAIGLLPAIRVYVNL 120
Y2b -------------------
N2b PFRISLRFSVAGAELAVLL 139
Supplementary Fig. 3 The 2b fusion proteins in the recombinant constructs used in this study. A, Constructions of A1Ds expressing the 2b-Ds-Red2 fusion protein. B, Alignment of the amino acid sequences of Y2b and the 2b protein of CMV-N (N2b). The C-terminal of N2b was coincidentally created by inserting a DNA fragment into the CMV-A1 vector. The nucleotide sequence of N2b was deposited to GenBank (accession no. LC167300).

## Slide 4
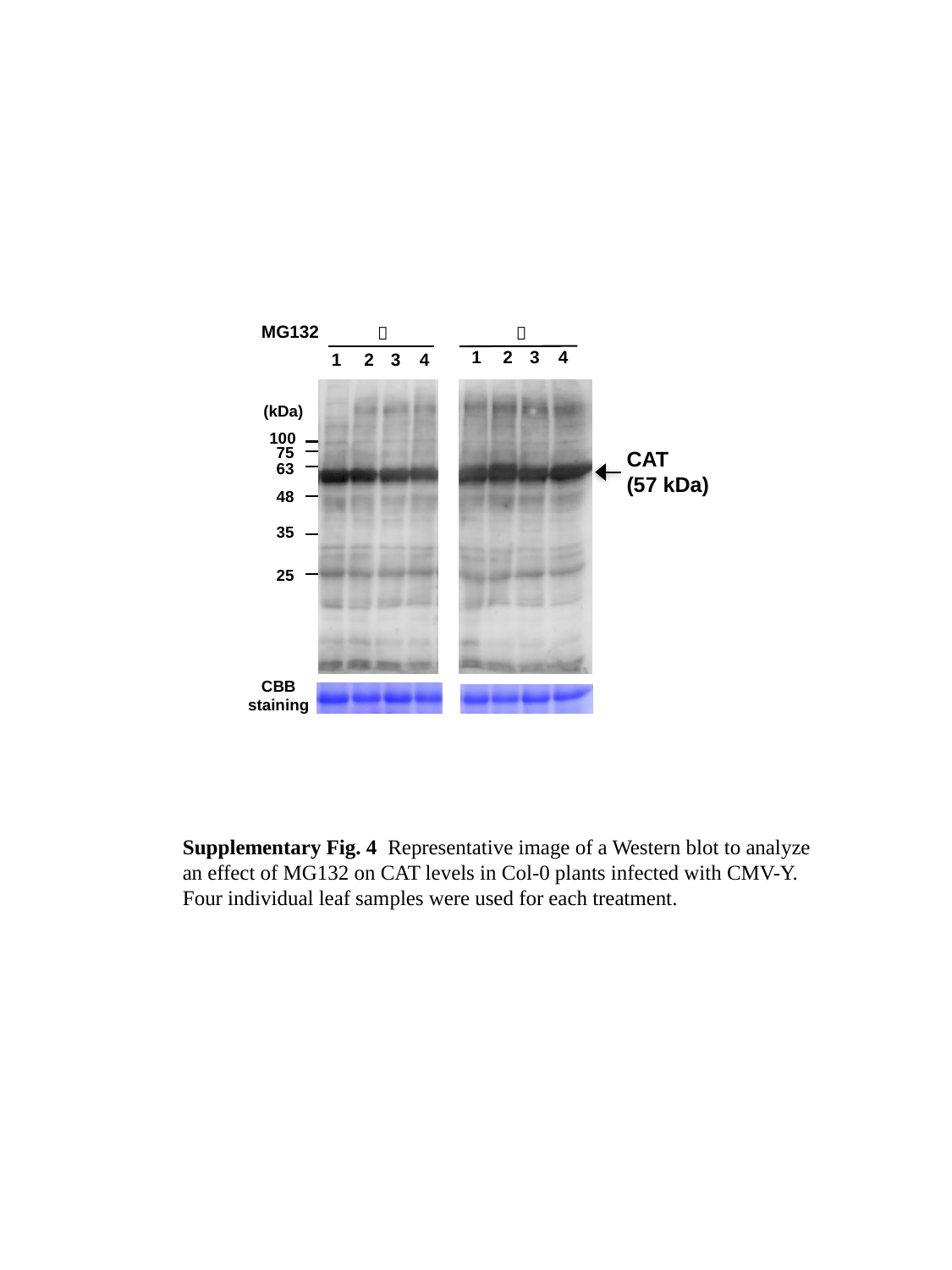

MG132
－
＋
4
1
2
3
CAT
(57 kDa)
4
1
2
3
(kDa)
100
75
63
48
35
25
CBB
staining
Supplementary Fig. 4 Representative image of a Western blot to analyze an effect of MG132 on CAT levels in Col-0 plants infected with CMV-Y. Four individual leaf samples were used for each treatment.
